# Analysis of temporal expression of HTLV-2 reveals similarities and functional differences from HTLV-1

**DOI:** 10.1186/1742-4690-8-S1-A192

**Published:** 2011-06-06

**Authors:** Cecilia Bender, Alessia Cotena, Paola Ronzi, Francesca Rende, Ilaria Cavallari, Claudio Casoli, Vincenzo Ciminale, Umberto Bertazzoni

**Affiliations:** 1 Department of Life and Reproduction Sciences, Section of Biology and Genetics, Università degli Studi di Verona, Verona 37134, Italy; 2 Department of Clinical Sciences, Università degli Studi di Milano, Milano, Italy; 3 Department of Oncology, Università degli Studi di Padova, Padova, Italy

## 

In the present study, we developed a robust splice site-specific real-time RT-PCR method to quantitate all HTLV-2 transcripts. Results of this analysis conducted on three different infected cell lines (HTLV-2A Mo-T , C344 and HTLV-2B BJAB-Gu) showed that the most abundant mRNA was gag/pol followed by the accessory transcript 1-3, coding for the p28 and for p22/p20 proteins. The third most abundant mRNA was tax/rex.

To investigate if different mRNAs produced by HTLV-2 are expressed at different levels upon viral reactivation, we studied the kinetics of viral expression in PBMCs from three subjects infected with HTLV-2B and cultured in vitro for 48 hours. The level of expression of the full length gag/pol transcript was the highest in all samples. The tax/rex mRNA was detected already at time zero and increased very rapidly following in vitro culture, reaching the highest copy number between zero and 2-4 hours. The minus-strand APH-2 mRNA, was expressed at high level. As observed in the infected cell lines, the 1-3 mRNA was expressed at high levels in all subjects. This finding is particularly intriguing, as it encodes two proteins that were shown to exert a powerful control on Tax and Rex function. This peculiar pattern of expression, which is in striking contrast with that of HTLV-1, might in part explain the differential pathogenicity of the two viruses.

